# High Doses of ANA12 Improve Phenobarbital Efficacy in a Model of Neonatal Post-Ischemic Seizures

**DOI:** 10.3390/ijms25031447

**Published:** 2024-01-24

**Authors:** Preeti Vyas, Ira Chaturvedi, Yun Hwang, Joseph Scafidi, Shilpa D. Kadam, Carl E. Stafstrom

**Affiliations:** 1 Department of Neurology, Johns Hopkins University School of Medicine, Baltimore, MD 21205, USA; pvyas2@jhmi.edu (P.V.); ichatur1@jhu.edu (I.C.); yunhwang@stanford.edu (Y.H.); scafidi@kennedykrieger.org (J.S.);; 2 Department of Neurology and Developmental Medicine, Kennedy Krieger Institute, Baltimore, MD 21205, USA; 3 Department of Neuroscience, Johns Hopkins University School of Medicine, Baltimore, MD 21205, USA

**Keywords:** hypoxic-ischemic encephalopathy, neonatal seizures, phenobarbital, ANA12, TrkB, KCC2

## Abstract

Phenobarbital (PB) remains the first-line medication for neonatal seizures. Yet, seizures in many newborns, particularly those associated with perinatal ischemia, are resistant to PB. Previous animal studies have shown that in postnatal day P7 mice pups with ischemic stroke induced by unilateral carotid ligation, the tyrosine receptor kinase B (TrkB) antagonist ANA12 (N-[2-[[(hexahydro-2-oxo-1H-azepin-3-yl)amino]carbonyl]phenyl]-benzo[b]thiophene-2-carboxamide, 5 mg/kg) improved the efficacy of PB in reducing seizure occurrence. To meet optimal standards of effectiveness, a wider range of ANA12 doses must be tested. Here, using the unilateral carotid ligation model, we tested the effectiveness of higher doses of ANA12 (10 and 20 mg/kg) on the ability of PB to reduce seizure burden, ameliorate cell death (assessed by Fluoro-Jade staining), and affect neurodevelopment (righting reflex, negative geotaxis test, open field test). We found that a single dose of ANA12 (10 or 20 mg/kg) given 1 h after unilateral carotid ligation in P7 pups reduced seizure burden and neocortical and striatal neuron death without impairing developmental reflexes. In conclusion, ANA12 at a range of doses (10–20 mg/kg) enhanced PB effectiveness for the treatment of perinatal ischemia-related seizures, suggesting that this agent might be a clinically safe and effective adjunctive agent for the treatment of pharmacoresistant neonatal seizures.

## 1. Introduction

Neonatal seizures remain a serious clinical challenge because they are difficult to diagnose and treat and are often associated with poor neurological outcomes [1]. Perinatal arterial ischemic stroke is one of the most common causes of seizures in term neonates, accounting for 7.5–20% of neonatal seizures. There is a major unmet need to identify an optimal anti-seizure medication (ASM) to treat neonatal seizures, as ~50% of babies do not respond to phenobarbital (PB), the current first-line agent [2,3,4]. Even with new insights into the role of Cl^−^ co-transporters in neonatal seizure susceptibility, there exists no approved mechanism-specific ASM in this age range. Therapeutic hypothermia, the current first-line non-pharmacologic treatment for hypoxic-ischemic encephalopathy (HIE), fails to reduce the seizure burden in neonates with severe HIE and fails to stop electrographic seizures in moderately severe HIE [5,6]. The KCC2 Cl^−^ co-transporter is the chief Cl^−^ extruder in neurons and KCC2 downregulation following ischemia severely impairs a cell’s ability to extrude Cl^−^. The resultant reversal of the transmembrane Cl^−^ gradient results in GABA-mediated depolarization instead of hyperpolarization and may lead to the emergence of pharmacoresistant seizures [7].

ANA12 (N-[2-[[(hexahydro-2-oxo-1H-azepin-3-yl)amino]carbonyl]phenyl]-benzo[b]thiophene-2-carboxamide) is a small-molecule TrkB (tyrosine receptor kinase B) receptor antagonist that transiently blocks BDNF (brain derived neurotrophic factor)-TrkB activation during the pathogenesis of HIE [8,9,10]. Recent research indicates the role of KCC2 as a potential target for multiple neurological disorders [11,12]. In a mouse model of pharmacoresistant neonatal seizures, ANA12, in combination with PB (25 mg/kg IP), rescued KCC2 expression and significantly reduced seizures in a dose-dependent manner [3]. A single intraperitoneal (IP) dose of 5 mg/kg of ANA12, in combination with 25 mg/kg PB IP, controlled seizures and restored KCC2 downregulation in post-ischemic P7 mice, with no apparent adverse effects [8,9,10]. These findings were further supported by similar rescue of seizures by small-molecules like CLP-290 that are putative KCC2 functional enhancers.

The present study aims to determine whether a higher dose of ANA12 and/or a lower dose of PB, compared to previously tested combinations, would improve anti-seizure efficacy. Achieving optimal seizure control with increased effectiveness is the most important objective in the treatment of neonatal seizures [13]. Our novel approach restores KCC2 function by modulating the BDNF-TrkB interaction-mediated KCC2 degradation in newborn mice with seizures caused by ischemia, providing a foundation for further studies to explore the link between neonatal seizures, pharmacoresistance, and the TrkB pathway. Rectifying this knowledge gap may help determine novel therapeutic targets for drug-resistant post-ischemic seizures.

## 2. Results

### 2.1. Antiseizure Efficacy of ANA12 in Combination with PB

**Figure 1:**
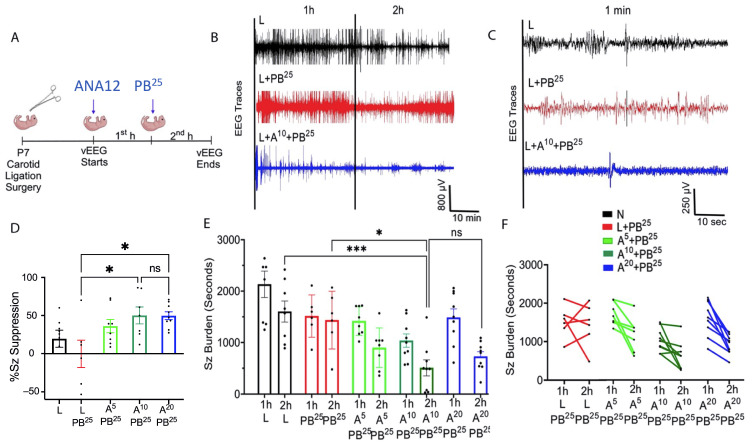
Antiseizure efficacy of ANA12 in combination with PB. (**A**) Experimental paradigm. ANA12 given after ligation followed by PB^25^ loading dose 1 h later. (**B**) Representative EEG traces (2 h) for the three treatment groups, with a vehicle injection administered after 1 h in those untreated (black trace). (**C**) Expanded time scale of a 1 min segment from each EEG in (B) from the midpoint of the second hour of EEG recording. (**D**) Graded doses of ANA12 + PB and ANA12 (20 mg/kg) + PB showed an increase in the percentage of seizure suppression with plateauing of efficacy with the ANA12 (20 mg/kg) dose. Data are represented as mean ± SD. (**E**) EEG seizure burdens for first and second hour for all treatment groups. (**F**) Individual EEG seizure burden changes in first hour vs. second hour shown for treatment groups in (**E**). The significance is ascertained as *** *p* < 0.001, * *p* < 0.05. ns (not significant), N (Naïve controls), L (Ligated only), A (ANA12), PB (Phenobarbital). All animals were ligated except group N.

ANA12 (10 mg/kg) improved PB efficacy and reduced the baseline seizure burden compared to ANA12 (5 mg/kg). No additional reduction of seizure burden was seen with the higher (20 mg/kg) ANA12 dose. The effect of acute ischemic seizures in mouse pups was assessed using continuous video-electroencephalogram (vEEG) recordings. Manifestations of seizure activity on EEG can range from electrographic-only seizures to prolonged ictal discharges. After unilateral carotid ligation, pups underwent 1 h of baseline vEEG recording. In our mouse model of unilateral carotid ligation (Figure 1A), pups presented with PB-resistant ischemic neonatal seizures at P7 (Figure 1B).

After 1 h of vEEG recording, pups received a loading dose of PB followed by a second hour of vEEG recording. Seizures in pups that only received a PB loading dose but no ANA12 (PB-only) were pharmacoresistant, as the seizures were suppressed for a short period and then the seizure burden remained high in the second hour. In pups pretreated with ANA12 (10 mg/kg) after ligation, the seizure baseline was dramatically reduced (508.9 ± 154.1 s) compared to L (1604 ± 204.9 s) and L + PB^25^ (1437 ± 229.3 s). Both ANA12 (10 mg/kg) + PB (50.11 ± 11.10%) and ANA12 (20 mg/kg) + PB (49.72 ± 15.48%) showed significantly higher % seizure suppression than L + PB. No significant difference was observed between ANA12 (10 mg/kg) + PB and ANA12 (20 mg/kg) + PB, which indicates a plateauing of the dose-response (Figure 1D–F).

### 2.2. Neurodevelopmental Reflex Tests

#### 2.2.1. Neonatal Tests

**Figure 2:**
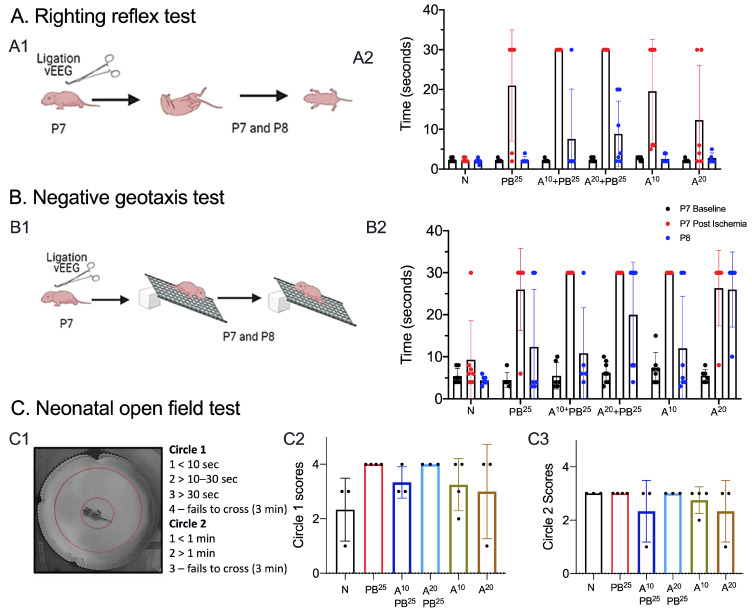
Neonatal neurobehavioral reflex tests as a biological marker of development. (**A**): (**A1**) Schematic of the righting reflex test conducted on P7 (before and after ligation) and on P8. (**A2**) Righting reflex test, with 30 s cutoff time to complete task. (**B**): (**B1**) Schematic of negative geotaxis test conducted on P7 before and after ligation, and on P8. (**B2**) Negative geotaxis test, with 30 s cutoff time to perform the task. Milestones for righting reflex and negative geotaxis scores were not significantly different in ligation-injured mice compared to naïve controls, irrespective of treatment. (**C**): (**C1**) Neonatal open field test on P8, with inner and outer circles highlighted in red. Open field scores for the inner Circle 1 (**C2**) and outer Circle 2 (**C3**) were not significant. No dose of ANA12 showed detrimental effects on neurodevelopmental reflexes in this mouse model of neonatal ischemia. N (Naïve controls), L (Ligated only), A (ANA12), PB (Phenobarbital). All animals were ligated except group N.

As shown in Figure 2, there were no detrimental effects of the tested doses of ANA12 on the righting reflex test or the negative geotaxis test. The results of both tests immediately after ischemia (P7) show prolonged latencies to righting and negative geotaxis, likely due to the sedated condition of the pups after receiving PB. However, on the negative geotaxis test, most mice did not recover to baseline by the following day (P8). In the open field test, there was no significant change in the distance traveled or the number of circle crossings for any treatment.

#### 2.2.2. Juvenile Tests

**Figure 3:**
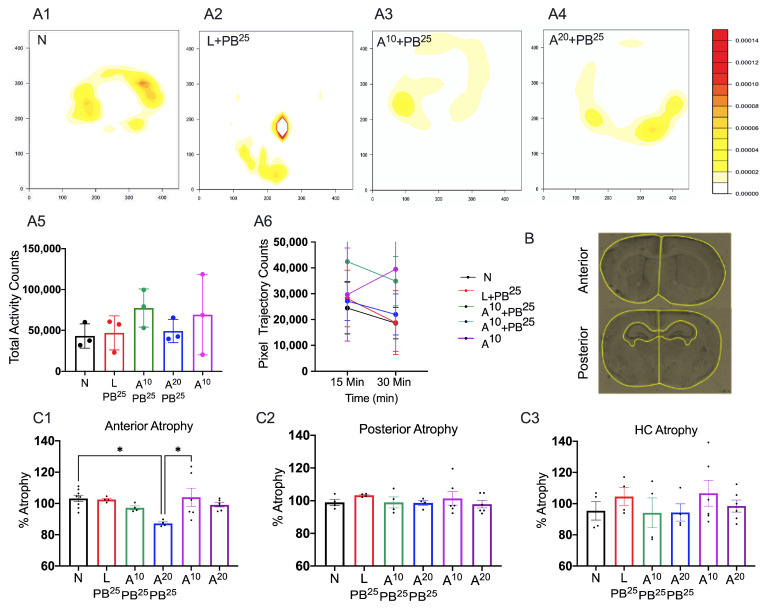
(**A1**–**A4**) Representative heat maps displaying movement activity of mice in the juvenile open field test. There was no difference in mice movement as a function of PB or ANA treatment. (**A5**) Total movement in open field expressed as pixels. (**A6**) The ability of mice to habituate to the chamber over 30 min in the open field. (**B**) Regions of interest on cresyl violet-stained P18 brain sections at 10×. Scale: 100 µm. (**C1**–**C3**) No atrophy detected except for in the highest dose tested in the anterior cerebrum with A^20^ compared to naïve controls (n = 6–8). The significance is ascertained as * *p* < 0.05. n (number of animals), N (Naïve controls), L (Ligated only), A (ANA12), PB (Phenobarbital). All animals were ligated except group N.

Mice from all groups gained weight similarly from P7 to P18 (Supplementary Figure S1). In the juvenile open field test, there was no difference in mice movement as a function of PB or ANA treatment (Figure 3(A1–A6)). On the hind-limb clasping test, there was no clasping of the hind limbs in any treatment group, and no detrimental effects of the tested doses of ANA12 were observed (Supplementary Figure S2).

Mice treated with ANA12 (20 mg/kg) had significantly more anterior cortical atrophy than N animals (Figure 3B,(C1)) but there was no difference across groups in posterior cortical or hippocampal atrophy (Figure 3(C2,C3)).

### 2.3. Fluoro-Jade Staining

**Figure 4:**
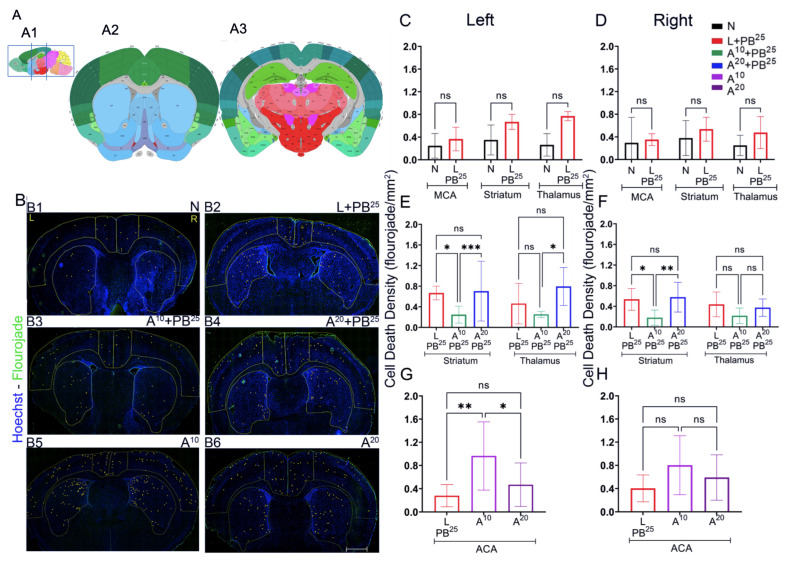
Fluoro-Jade (FJ) staining as a function of neuronal cell death associated with unilateral ligation. (**A**) Regions of interest (ROIs) in brains harvested 24 h post-ischemia. Boxed areas (**A1**) represent brain sections analyzed with ROIs (**A2**,**A3**), marked in yellow. (Allen Mouse Brain Atlas, mouse.brain-map.org and atlas.brain-map.org, accessed on 4 March 2022). (**B**): (**B1**–**B6**) Pictographs showing Fluoro-Jade positive cells (marked in yellow) in anterior sections. (**C**–**H**) No statistical differences were observed in either hemisphere for any ROIs in naïve vs. L + PB^25^. Scale bar: 500 µm (**C**,**D**). Significant reduction in cell death in all brain regions (except right thalamus) was observed in A^10^ + PB^25^ treated mice (**E**,**F**). Fluoro-Jade positive cells were increased in right cortex and left striatum in anterior and posterior brain sections after right unilateral ligation. A^10^ and A^20^ alone showed an increase in cell death vs. L + PB^25^ in the left anterior cerebral artery (left ACA) distribution (**G**,**H**). A^10^ + PB^25^ decreased cell death in the striatum and thalamus, also represented as fewer Fluoro-Jade positive cells in A^10^ + PB^25^ ((**B3**) vs. (**B2**–**B6**)). A^20^ + PB^25^ showed no decrease in cell death vs. L + PB^25^. The significance is ascertained as *** *p* < 0.001, ** *p* < 0.01, * *p* < 0.05. ns (not significant), N (Naïve controls), L (Ligated only), A (ANA12), PB (Phenobarbital), ACA (anterior cerebral artery), MCA (middle cerebral artery). All animals were ligated except group N.

We quantified cell death in the ischemic mouse model using the Fluoro-Jade stain (Figure 4). Brains were harvested 24 h after ischemia. Anterior and posterior coronal brain sections have ROIs highlighted in yellow (Figure 4A). We observed that ANA12 (10 mg/kg) + PB^25^ reduced neuronal cell death in the right cortex (the ligated side) in anterior brain sections, demonstrating a neuroprotective effect.

### 2.4. Anti-Seizure Potential of ANA12 (10 mg/kg IP) + PB (15 mg/kg IP)

**Figure 5:**
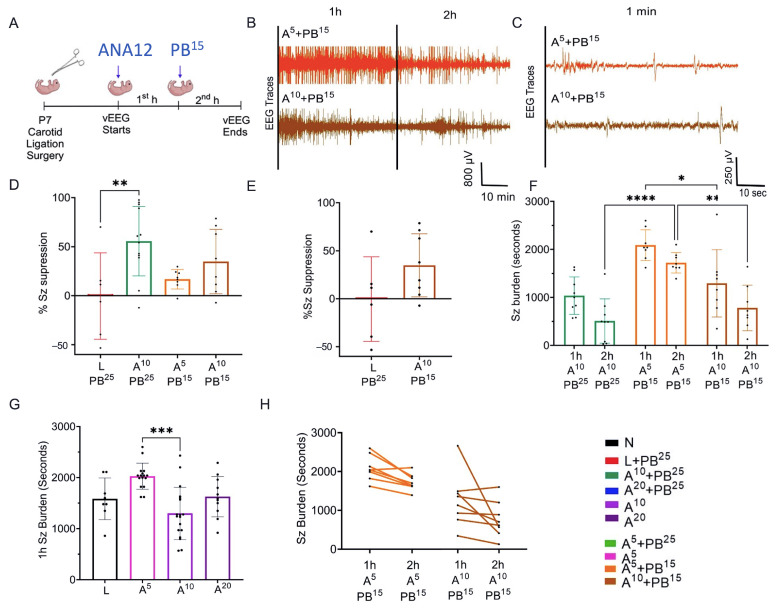
Anti-seizure potential of ANA12 and PB. (**A**) Experimental paradigm: A^5^ or A^10^ administered post-ligation followed by a PB^15^ dose 1 h later. (**B**) EEG 2 h and (**C**) 1 min traces for A^5^ + PB^15^ and A^10^ + PB^15^, (**D**,**E**). A^10^ + PB^25^ (but not A^5^ + PB^15^ and A^10^ + PB^15^) showed significantly higher % seizure suppression vs. L + PB^25^. (**F**) The second hour after A^5^ + PB^15^ showed significantly higher seizure burden than the second hour A^10^ + PB^25^; no significant difference A^10^ + PB^25^ vs. A^10^ + PB^15^. (**G**) First hour seizure burden for three doses of ANA12, (**H**) individual second hour seizure burden ANA12 (5 mg/kg) + PB^15^ and ANA12 (10 mg/kg) + PB^15^ (**H**). A^5^ + PB^15^ treated group had higher first hour seizure burden vs. A^10^ + PB^15^. A^10^ + PB^15^ lower seizure baseline vs. A^5^ + PB^15^. The significance is ascertained as **** *p* < 0.0001, *** *p* < 0.001, ** *p* < 0.01, * *p* < 0.05. N (Naïve controls), L (Ligated only), A (ANA12), PB (Phenobarbital). All animals were ligated except group N.

Considering the potential adverse effects associated with PB, we next treated the P7 pups with the optimal dose of ANA12 (10 mg/kg) in combination with a lower PB dose than the usual standard PB loading dose of 25 mg/kg IP. EEG was recorded to see whether decreasing the PB loading dose to 15 mg/kg IP would still decrease seizure burden. The EEG traces in Figure 5B,C show that A^10^ + PB^15^ had a lower seizure burden at baseline during the first hour and both groups (A^5^ + PB^15^ and A^10^ + PB^15^) showed a decrease in seizure burden during the second hour. However, we observed no significant enhancement of seizure suppression with ANA12 (10 mg/kg) + PB^15^ in comparison with PB^25^ monotherapy. Interestingly, A^10^ + PB^25^ showed significantly more seizure suppression than PB alone.

## 3. Discussion

The results presented here suggest that single dose interventions of a small-molecule TrkB antagonist as adjunct treatment with PB can significantly rescue PB-resistant seizures in a well-characterized model of neonatal HIE seizures. This rescue was dose-sensitive with a plateauing effect for efficacy at the 2× higher doses of ANA12 tested, which may indicate limitations of the small-molecule related to binding and CNS bioavailability that could result in off-target effects. In the future, such limitations could be addressed using standard drug-development strategies. The neuroprotective effect of the most efficacious anti-seizure dose of ANA12 with no significant detrimental effects noted on neurodevelopmental reflexes and weight gain provide promising support for this novel acute intervention strategy. The clinical focus, in addition to treating presenting symptoms associated with ischemic insults, is also to protect the brain during the acute excitotoxic crisis. Hence, in addition to curbing seizures, the management goals now extend to preventing significant long-term comorbidities. This strategy is even more critical for a fragile neonatal population where such an event affects the entire developmental trajectory and quality of life as is reported for HIE.

The novel ANA12 + PB combination therapy studied here is expected to significantly improve the efficacy over PB monotherapy in neonates as well as improve survival. The combination therapy suggests that the brain damage caused by seizures is reduced and that neurocognitive outcome is improved in HIE neonatal pups. This novel intervention strategy with the ANA12 + PB combination is designed for new formulations with the intent to seamlessly integrate with existing newborn intensive care unit (NICU) clinical practice, including the common use of therapeutic hypothermia (TH). The salient findings presented here are that a single dose of ANA12 (10 mg/kg) (1) improved PB efficacy and reduced baseline EEG seizure burden in comparison with lower doses; (2) caused no detrimental effects on selected neurodevelopmental reflexes or weight gain; (3) reduced neuronal cell death in the right anterior cortex, demonstrating a neuroprotective effect. A higher dose of ANA12 (20 mg/kg) + PB (4) afforded no further anti-seizure efficacy and increased atrophy in anterior cortical regions, and (5) reducing the PB dose from 25 to 15 mg/kg did not afford sufficient seizure control, indicating the importance of using the standard PB loading dose in the model and for HIE in general.

The strategies used in this study are novel and depend on TrkB modulation of KCC2 at the membrane level. Developmentally, KCC2 expression in neurons is tightly regulated and phosphorylation sites that direct membrane insertion are also age-dependent [14]. Therefore, one limitation of this strategy may be that it might not be applicable to premature infants with seizures due to developmentally low levels of KCC2 in the neocortex at such early stages. However, for full-term infants with other neonatal seizure etiologies and encephalopathies due to genetic causes, this KCC2-targeted strategy may be feasible for the treatment of early life refractory seizures [15]. Other novel strategies targeting TrkB in preclinical epilepsy research strengthen this approach to neonatal seizure treatment [16,17]. The pharmacological advantage of combination therapies with GABAergic agents in general, and similar to the add-on PB strategy investigated in this study, is the potential enhancement of Cl^−^ extrusion capacity of KCC2 in neurons. This would result in lower intracellular Cl^−^ levels, allowing endogenous GABA to elicit stronger hyperpolarization and efficiently reduce the synchronous neuronal firing associated with seizures [18].

Several reports have documented mutations in the neuron-specific type 2 K^+^/Cl^−^ cotransporter KCC2 as a cause of early-life seizures [19] and epilepsy. KCC2 is the chief chloride extruder in neurons and is crucial for chloride homeostasis during neural development and in hyperexcitable states. KCC2 is essential for the establishment of normal brain function; it determines the functioning of the glycine and GABA_A_ receptors that serve as crucial targets for several ASMs [20]. Upstream and downstream mediators of KCC2 functional modulation are promising antiseizure targets [21], but questions about clinical efficacy and toxicity remain. Therefore, we developed a novel treatment protocol for HIE-induced refractory seizures in neonates. We previously demonstrated that ANA12 restores post-ischemic KCC2 downregulation, as an adjunct to the first-line ASM, phenobarbital (PB) [3]. We are developing a novel treatment protocol utilizing ANA12 + PB for HIE-induced seizures in neonates. We and several other groups have shown that ischemia results in KCC2 downregulation. The neonatal brain naturally has a lower expression of neuron-specific KCC2, which is known to be developmentally regulated. KCC2 hypofunction results in decreased inhibition and increased network hyperexcitability that underlies numerous disease states including epilepsy, neuropathic pain, and neuropsychiatric disorders [22,23]. The holy grail of KCC2 cotransporter biology is to identify ways to restore KCC2 normal function to restore physiological functional levels of synaptic inhibition and neuronal network activity. We have shown that KCC2 downregulation can be prevented by acutely blocking TrkB activation with ANA12. ANA12, currently labeled for research use only (RUO), is available from chemical providers such as Millipore Sigma (Burlington, MA, USA). It is a known selective, non-competitive antagonist of TrkB, the main receptor of brain-derived neurotrophic factor. In mice, ANA12 crosses the blood–brain barrier and exerts a central TrkB blockade, producing effects as early as 30 min and as long as 6 h following intraperitoneal injection.

TrkB antagonists like ANA12 have never been used as a class of drugs in humans. Since this novel small molecule is blood–brain-barrier permeable, we expect brain availability in humans after IV delivery to be optimal. Our intervention is targeted at a clinically feasible window following the detection of HIE seizures in neonates and therefore fits well with the current standard treatment protocols. This is critical since pretreatment with novel drugs is not a practical option in non-symptomatic neonates. Currently, PB is administered by IV infusion in the NICU. Our novel treatment can add an appropriate optimal and non-toxic dose of ANA12 solution to the PB infusion at the time of first treatment intervention when HIE-related seizures are confirmed by EEG in at-risk babies. Our study generated key proof-of-concept data to support the development of ANA12 + PB to treat neonatal seizures. We demonstrated the anti-seizure efficacy of ANA12 + PB and measured the ability of the injected neonates to thrive by comparing three different doses of ANA12. We tested the safety of the highest safe dose of ANA12 in combination with PB to additionally investigate the safety of the combination. We have not observed apparent murine toxicity to date with our optimal doses.

Overall, this study reports the successful rescue of PB resistance in a model of neonatal ischemic seizures with higher doses of ANA12 than previously reported [3,9,10]. Further expansion of this strategy could be applicable to other refractory early-life epilepsies and acute life-threatening seizures, such as status epilepticus, which are known to become refractory to first-line GABAergic ASMs. Combination therapeutic strategies like the one proposed here could provide additional tools for clinical management. Future advances in KCC2 pharmacology could help bolster the strategy proposed here with ANA12 + PB combination therapy. However, targeted therapeutics that would directly enhance KCC2 activity carry the risk of excessive KCC2 activity in acute emergency situations, which can be detrimental to the neonatal brain. High doses of PB are already known to have detrimental effects on long-term neurodevelopment; hence, new and safe therapies remain an unmet clinical need for HIE. Based on our current findings, we propose that this new interventional strategy is well targeted at a clinically feasible window with minimal toxicity in mice pups following the detection of HIE-associated neonatal seizures and therefore fits well with current standard treatment protocols. Once approved, ANA12 + PB will likely also be used off label to improve outcomes in indications where GABAergic drugs, when traditionally utilized as a monotherapy, remain seizure-refractory in subset populations of patients.

## 4. Materials and Methods

### 4.1. Animals

All experimental procedures and protocols were conducted in compliance with the guidelines of the Committee on the Ethics of Animal Experiments and were approved by the Animal Care and Use of Committee of Johns Hopkins University. Newborn CD-1 mouse litters (n = 10) were purchased from Charles River Laboratories (Wilmington, MA, USA) and arrived at P3–4 along with a dam. Mice were allowed to acclimate for 3–4 days before undergoing an ischemic insult. Equivalent numbers of male and female pups were used. Mice were housed on a 12 h light–dark cycle with food and water provided ad libitum.

### 4.2. Ischemic Insult

We employed standard surgical procedures as previously reported [9]. On P7, under isoflurane anesthesia, pups underwent permanent unilateral ligation of the right common carotid artery using 6-0 Surgisilk (Fine Science Tools, North Vancouver, BC, Canada). The outer skin was sutured with 6-0 monofilament nylon (Covidien, Mansfield, MA, USA) with additional local lidocaine anesthesia.

### 4.3. Sub-Dermal EEG Electrode Implantation and EEG Recordings

Immediately after ligation on P7, pups were implanted with subdermal EEG scalp electrodes (IVES EEG; Model # SWE-L25, Newburyport, MA, USA), 1 recording and 1 reference, overlying the left/right parietal cortices, with 1 ground electrode over the rostrum. EEG recordings were acquired using Sirenia Acquisition software (version 2.2.11) with synchronous video capture (Pinnacle Technology, Lawrence, KS, USA).

Data were acquired with sampling rates of 400 Hz with preamplifier gain of 100 and 0.5 Hz high-pass and 50 Hz low-pass filters. Data were scored by binning EEG in 10 s epochs. Similar to previous studies from our laboratory [3], seizures were defined as electrographic ictal events that consisted of high-amplitude spikes with a peak frequency of ≥7–8 Hz as detected by automated power spectrum analysis. These peaks lasted ≤ 6 s, which is longer than half of each 10 s epoch. Seizure burden was quantified by calculating the maximum percentage of each hour of recording that contained electrographic seizures. The mean ictal events and their durations were considered in seizure burden calculations.

After ligation, ANA12 was injected and baseline EEG was recorded for 1 h, after which PB was administered and EEG was recorded for another 1 h. One hour after the PB injection, electrodes were removed and the pups were returned to the dam.

### 4.4. Drugs and Treatment Groups

PB was prepared in pyrogen-free normal saline for all experiments. In initial experiments, ANA12 (10 mg/kg) was prepared in 5% DMSO, but the drug-vehicle solubility testing phase revealed that 5% DMSO was not ideal for higher doses of ANA12. Therefore, the vehicle was changed to 100% N-methyl-2-pyrrolidone (NMP), which is more compatible with ANA12. All drugs were freshly prepared on the day of the experiment and were injected IP using a Hamilton syringe. The drug treatment groups were designated as N, naïve controls; L, ligated only; A^5^, received 5 mg/kg ANA12 IP; A^10^, received 10 mg/kg ANA12 IP; A^20^, received 20 mg/kg IP; PB^15^, received 15 mg/kg PB IP; PB^25^, received 25 mg/kg PB IP. All animals were ligated except group N. All reagents used in the experiments were analytical grade. Distilled water was used throughout the experiments, wherever required.

### 4.5. Weights

Pups were weighed on P7 before ligation surgery, and then at P8, P10, P14, and P18 to monitor growth.

### 4.6. Neurobehavioral Tests

#### 4.6.1. Neonatal Neurobehavioral Tests (P7 and P8)

We tested neurobehavioral reflexes that correlate with developmental milestones in mice and serve as an early assessment of newborn neurologic function [24]. P7–P10 rodent pups are equivalent to near-term humans in terms of several markers of brain development, and newborn pups are too immature to undergo complex motor, sensory, and cognitive tests [7,25]. For example, pups’ eyes are not open before P10 and they do not have sufficient fur to thermoregulate. Therefore, we selected the following behavioral tests to assess brain and physical development, correlated the behavioral results with EEG findings, and assessed for drug toxicity.

- Righting reflex test

A pup is held firmly in the supine position, with all four paws upright. When released, time to righting is recorded (time to flip/roll over onto all four paws, with each paw perpendicular to the body). Each pup is allowed a maximum of 30 s to achieve this goal. Righting is scored as 0, lying on the back (or 30 s for the maximum allocated time); 1, lying on the left or right side or righting but in the wrong posture (or 30 s for the maximum allocated time); 2, successful righting with appropriate posture.

- Negative geotaxis test

A pup is placed on a grid facing down a 45-degree slope. A normal response is to turn and face up the slope. The test is passed when a pup turns up the slope. A cutoff time of 30 s is allowed to achieve the goal. Pups that fell or failed to turn upwards were scored zero. Occasionally, a pup would roll down the incline due to sleepiness rather than weakness. To avoid this confound, we performed two trials a day at 8 a.m. and 5:30 p.m. on P7 and P8.

- Neonatal open field test

On P8, pups were placed in the neonatal open field for 3 min and video-recorded (Figure 2). The test was scored based on the time spent in the inner circle (circle 1) versus time spent in the outer circle (circle 2). Scoring: Circle1 scores: 1 for <10 s, 2 for >10–20 s, 3 for >30 s, and 4 for failing to cross circle 1 in 3 min. Circle 2 scores: 1 for <1 min, 2 for >1 min, and 3 for failing to cross circle 2 in 3 min.

#### 4.6.2. Juvenile Neurobehavioral Tests (P18)

We again assessed neurobehavior in mice pups and carried out juvenile open-field testing for comparison with the neonatal phenotype. We also assessed hind-limb clasping, which is a marker of disease progression in several mouse models of neurodegeneration [26]. These phenotypes are observed in mice with cerebellum, basal ganglia, and neocortex lesions. The underlying mechanism can include cerebellar–cortical–reticular and cortico–striato–pallido–reticular pathways, possibly triggered by changes in noradrenaline and serotonin transmission [27].

- Juvenile open field test

This procedure was carried out in a square open-field chamber (40.6 × 40.6 cm, Accuscan, Columbus, OH, USA) mounted within sound-attenuating shells. Behavior was monitored via a grid of invisible infrared light beams mounted on the sides of the walls of the arena. Data were collected and analyzed using VersaMax Analyzer software version 4.0 (Accuscan, Columbus, OH, USA). To examine activity levels and habituation, mice were exposed to the test chambers for 30 min on each of two consecutive days beginning at P18. To begin a session, a mouse was placed in the center of the chamber and allowed to move about freely for 20 min. The arena was cleaned with 70% ethanol after each mouse completed a session.

- Hind-limb clasping

Each mouse was suspended by the tail and observed for 30 s and its ability to clasp with hind limbs was assessed.

### 4.7. Staining and Image Analysis

To assess cell death, Fluoro-Jade C (FJC) staining was performed using the FJC Ready-to-Dilute Staining Kit (Biosensis, Thebarton, South Australia, Australia). Briefly, the brain was sectioned using a cryostat maintained at minus 20 °C. The brain was mounted using the cryo-embedding matrix OCT, each section with a thickness of 20 microns. These slides with sagittal brain sections were immersed in 1% sodium hydroxide solution for 5 min, then rinsed in 70% ethanol, and then distilled water for 2 min each. Slides were then incubated in 0.06% potassium permanganate solution for 10 min, followed by rinsing for 2 min in distilled water, and then transferred into a 0.0001% solution of FJC (Millipore, Burlington, MA, USA) for 10 min. Slides were washed with distilled water three times for 1 minute each, dried in an incubator at 50 °C for 5 min, then cleared in xylene for 5 min and covered with Prolong gold antifade mounting media (Thermofisher Scientific, Waltham, MA, USA).

Sections were imaged and analyzed using a fluorescence microscope, Leica DMi8 (Leica Microsystems, Wetzlar, Germany) equipped with Leica Application Suite software (LASX office 1.4.4, (Leica Microsystems, Wetzlar, Germany)). Using ImageJ software (version 1.6.0), regions of interest (ROIs) for anterior and posterior brain sections of the treatment groups were selected. The ROIs for frontal brain sections were left and right parasagittal cortex, somatosensory cortex, and striatum, whereas the ROIs for parietal brain sections were left and right parasagittal cortex, somatosensory cortex, striatum, hippocampus, and thalamus. FJC-positive cells were counted, and the areas of cell death were exported in pixels and then converted to mm^2^ using a scale factor of 10.82. The density of degenerated neurons/mm^2^ in each region for the treatment group was compared.

For brain atrophy, we placed the slides in cresyl violet acetate solution for 5 min. Slides were rinsed with three changes of distilled water and then dehydrated in graded concentrations of alcohol. We then cleared the slides with xylene and mounted them using a DPX mounting medium (a mixture of dysterene and xylene, Sigma-Aldrich, St. Louis, MO, USA) for bright field microscopy.

### 4.8. Statistical Analysis

For all EEG experiments, data quantification and analysis were performed blinded to the genotype, sex, and treatment condition. All statistical tests were performed using GraphPad Prism software (version 8.4.0). A two-way analysis of variance (ANOVA) was performed with Tukey’s post hoc correction. One-way ANOVA was performed with Dunnett’s post hoc correction. Paired and unpaired *t*-tests were two-tailed. Survival analysis was performed by a Mantel–Cox test. Data are represented as bar graphs representing the mean, with 385 dot plots representing each data point. All errors bars are ± standard deviation. *p* values of ≤0.05 are considered statistically significant.

## Data Availability

Raw data sharing with the scientific community will be handled under the auspices of the PI (S.D.K.). Following publication, the preclinical EEG data are archived for access to any future interested collaborators.

## Supplementary Materials

The following supporting information can be downloaded at: https://www.mdpi.com/article/10.3390/ijms25031447/s1.
