# Supplementary material for: High Doses of ANA12 Improve Phenobarbital Efficacy in a Model of Neonatal Post-Ischemic Seizures
Source: Int J Mol Sci. 2024 Jan 24;25(3):1447. doi: 10.3390/ijms25031447 (PMC10855037; doi:10.3390/ijms25031447)
Supplement: Supplementary file 1 [file ijms-25-01447-s001.zip › ijms-2773563-supplementary.pdf]

Supplementary Materials: The following supporting information can be downloaded at: https://www.mdpi.com/article/10.3390/ijms25031447/s1, Figure S1: Weights; Figure S2: Hind-limb Clasping Test.

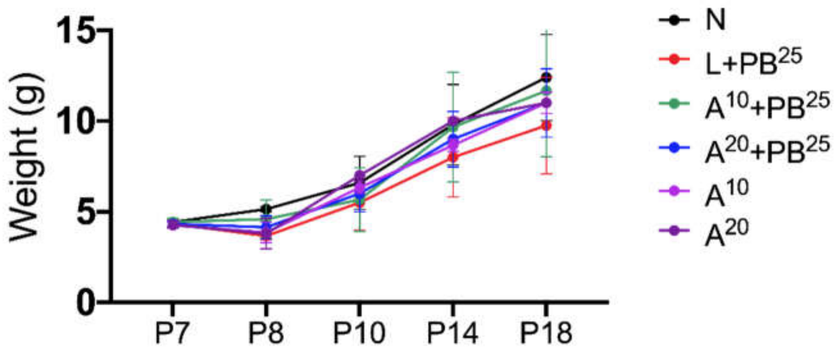

Supplementary Figure S1. Weights. Weights (g) monitored from P7 to P18 for both female and male mice. No treatment groups showed any deficit in their ability to gain weight.

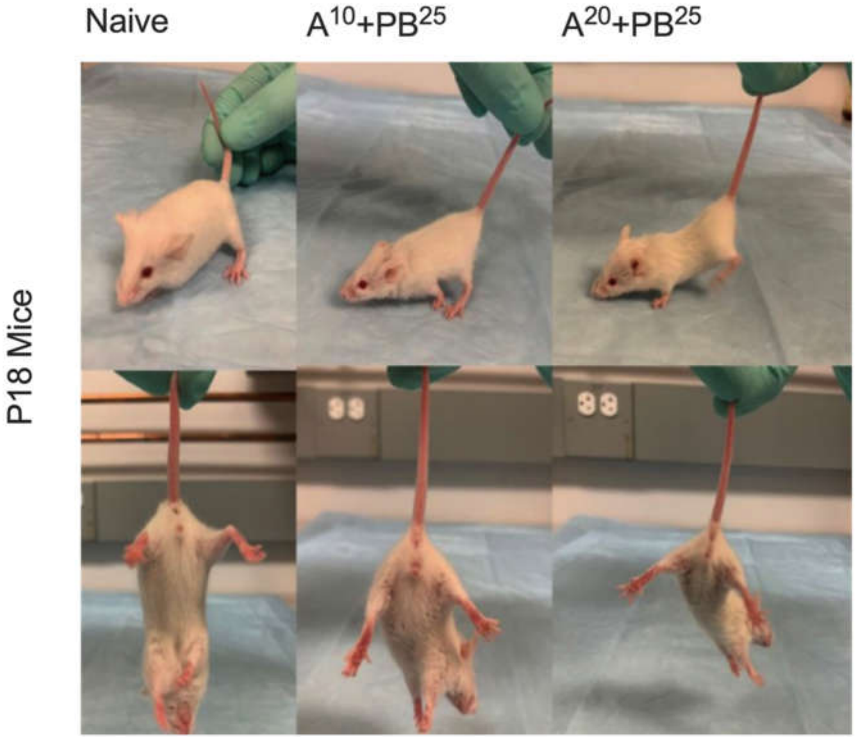

Supplementary Figure S2. Hind-limb Clasping Test. Hind-limb clasping test for graded doses of ANA12+PB^{25} compared to the naïve. The treated animals showed no hind limb clasping. No significant difference in activity of the naïve or control groups were seen compared to graded doses of ANA12 with or without the addition of PB^{25}.
